# Coordination of hypoxia adaptation and iron homeostasis in human pathogenic fungi

**DOI:** 10.3389/fmicb.2012.00381

**Published:** 2012-11-06

**Authors:** Dawoon Chung, Hubertus Haas, Robert A. Cramer

**Affiliations:** ^1^Department of Microbiology and Immunology, Geisel School of Medicine at DartmouthHanover, NH, USA; ^2^Division of Molecular Biology, Biocenter, Innsbruck Medical UniversityInnsbruck, Austria

**Keywords:** hypoxia, iron homeostasis, pathogenic fungi, SREBPs, GATA factor, CBC-binding factor, heme, ergosterol biosynthesis

## Abstract

In mammals, hypoxia causes facilitated erythropoiesis that requires increased iron availability with established links between oxygen and iron in regulation of the transcription factor hypoxia-inducible factor. Therefore, cellular responses to hypoxia and iron starvation are linked in mammals and are host conditions that pathogens encounter during infection. In human pathogenic fungi, molecular mechanisms underlying hypoxia adaptation and iron homeostasis have been investigated. However, the interconnected regulation of hypoxia adaptation and iron homeostasis remains to be fully elucidated. This review discusses the potential transcriptional regulatory links between hypoxia adaptation and iron homeostasis in human pathogenic fungi. Transcriptome analyses demonstrate that core regulators of hypoxia adaptation and iron homeostasis are involved in regulation of several common genes responsible for iron acquisition and ergosterol biosynthesis. Importantly, iron starvation increases susceptibility of fungal cells to antifungal drugs and decreased levels of ergosterol, while key hypoxia regulators are also involved in responses to antifungal drugs and mediating ergosterol levels. We suggest that pathogenic fungi have developed a coordinated regulatory system in response to hypoxia and iron starvation through (i) regulation of expression of hypoxia-responsive and iron-responsive genes via cross-linked key regulators, and/or (ii) regulation of factors involved in ergosterol biosynthesis. Thus, both oxygen and iron availability are intimately tied with fungal virulence and responses to existing therapeutics and further elucidation of their interrelationship should have significant clinical implications.

## INTRODUCTION

Invasive fungal infections (IFIs) including candidiasis, aspergillosis, cryptococcal meningitis, zygomycosis, and endemic mycoses have become a significant threat to immunocompromised and immunocompetent individuals. Treatment options for IFIs are limited due to host toxicity issues, lack of drug targets, and emerging drug resistance (Latge and Calderone, 2002; Thompson and Patterson, 2008; Denning and Hope, 2010). Importantly, the recent rise in the use of medical procedures requiring immunosuppressive therapies adds concerns for exacerbating the current impact of IFIs on human wellbeing (Chayakulkeeree et al., 2006; Denning and Hope, 2010; Kontoyiannis et al., 2010; Pappas et al., 2010). Given the rapid technological advances in medicine and increased human life-span it seems clear that life threatening fungal infections will continue to be a major clinical problem for the foreseeable future.

Like other diseases, development of fungal disease is a constant battle between the pathogen and host. To “win” the battle and survive, fungi must tolerate and overcome diverse *in vivo* microenvironmental stress conditions during infection. Host microenvironmental parameters that can affect the ability of fungi to cause disease include temperature, pH, carbon and nitrogen sources, iron acquisition, and gas tension (carbon dioxide and oxygen levels) among others (Askew, 2008; Cooney and Klein, 2008; Dagenais and Keller, 2009; Wezensky and Cramer, 2011). In this review, we focus on how fungal responses to hypoxia (significantly low levels of oxygen) and iron limitation may be interconnected (Weinberg, 1999a; Schaible and Kaufmann, 2004; Cramer et al., 2009; Salahudeen and Bruick, 2009; Wezensky and Cramer, 2011). Both of these stresses have been observed to occur during fungal pathogenesis, and fungal responses to them have been associated with virulence and currently used antifungal drugs.

Due to the involvement of oxygen in iron metabolism (e.g., oxidation of Fe^2+^ to Fe^3+^ for iron storage; Arosio et al., 2009) and iron requirements for oxygen transport or respiration (e.g., heme cofactors; Goldberg et al., 1988), the presence of integrated regulation of iron homeostasis and hypoxia adaptation has been hypothesized. Oxygen levels in healthy human tissues are 20–70 mmHg (2.5–9% O_2_), and damage or inflammation often causes hypoxic environments in the tissues with an oxygen level of less than 10 mmHg (~1% O_2_; Lewis et al., 1999). In healthy tissue and fluids, the concentration of free iron is extremely low (10^-24^ ~ 10^-18^M; Bullen et al., 1978, 2005; Martin et al., 1987), and it has been reported that serum iron levels decrease further by fever during infection (Kluger and Rothenburg, 1979). These data suggest that both hypoxia and iron limitation are natural defense mechanisms of mammalian hosts against microbial infection.

In response to hypoxia, mammalian cells attempt to increase oxygen uptake/utilization by enhancing red blood cell production (erythropoiesis; Goldberg et al., 1988). Erythropoiesis involves hemoglobin whose structure contains heme. In order to induce erythropoiesis in hypoxia, cells increase iron availability to support an increased demand for heme biosynthesis. Thus, in mammals, the cellular responses to hypoxia or iron starvation might lead to similar consequences such as improvement of iron availability (Chepelev and Willmore, 2011). Whether similar mechanisms exist in fungi remains to be fully elucidated.

Studies on hypoxia-inducible factor-1 (HIF-1) in mammals and *Caenorhabditis elegans* have elucidated a regulatory link in cellular responses to hypoxia and iron limitation (Mendel, 1961; Rolfs et al., 1997; Yoon et al., 2006; Peyssonnaux et al., 2008; Salahudeen and Bruick, 2009; Baek et al., 2011; Chepelev and Willmore, 2011; Romney et al., 2011). Stabilization of HIF-1 is induced in response to hypoxia and the presence of microbial pathogens, and HIF-1 plays a role in adaptation of stress environments and the innate immune system (Nizet and Johnson, 2009). HIF is post-translationally regulated by oxygen via hydroxylation of a regulatory subunit, HIF-α (Wang and Semenza, 1993; Poellinger and Johnson, 2004). This process is mediated by prolyl-hydroxylases (PHDs) that require iron as a cofactor (Appelhoff et al., 2004). The promoter sequence of the gene encoding the iron transport protein transferrin (Tf) contains HIF-1 binding sites and expression of Tf increases in hypoxia due to induced HIF-1 expression (Rolfs et al., 1997). An iron response element (IRE) is found in the promoter sequence of HIF-2α, which implies that induction of HIF and resulting hypoxia adaptation is regulated in part by iron availability (Ozer and Bruick, 2007; Sanchez et al., 2007; Salahudeen and Bruick, 2009). In *C. elegans*, expression of two genes, *ftn-1* and *ftn-2*, encoding the iron-storage protein ferritin is regulated by HIF-1 binding of hypoxia response element (HRE) in the promoters of *ftn-1* and *ftn-2* (Romney et al., 2011). Currently, no HIF-1 homolog has been identified in fungi. Given our increasing understanding of fungal responses to hypoxia and iron limitation and their clinical relevance, it is important to uncover and define regulation mechanisms of fungal hypoxia adaptation and iron homeostasis. In this review, we will describe potential regulatory mechanisms between iron homeostasis and hypoxia adaptation in fungi based on research mainly in three important pathogenic fungi,* Candida albicans*, *Cryptococcus neoformans*, and *Aspergillus fumigatus*.

## FUNGAL HYPOXIA AND IRON STRESS RESPONSES – TRANSCRIPTIONAL REGULATORS

Much of our fungal hypoxia and iron responses knowledge comes from studies on global transcriptional regulators that mediate adaptation to hypoxia and low-iron environments and these are the focus of this review. Phenotypes of gene deletion mutants of the major transcriptional regulators are summarized in **Tables 1 and 2**.

**TABLE 1:** Phenotypes of key regulatory gene null mutants for hypoxia adaptation.

Organism	Regulator	Phenotype of knock-out mutants	Virulence-related	Reference
*S. pombe*	Sre1	- defective growth under anaerobic conditions	NA	Hughes et al. (2005)
*C. neoformans*	Sre1	- defective growth under hypoxia and iron starvation - hypersusceptibility to azole drugs - reduced ergosterol synthesis in normoxia and hypoxia	Yes	Chang et al. (2007), 2009
*A. fumigatus*	SrbA	- defective growth in hypoxia and iron starvation - hypersusceptibility to triazole drugs - defective cell polarity and ergosterol synthesis - decreased siderophore production in iron starvation	Yes	Willger et al. (2008), Blatzer et al. (2011)
*C. albicans*	Upc2	- defective growth and hyphal formation in hypoxia - reduced ergosterol synthesis - hypersusceptibility to azole drugs	NA	Silver et al. (2004), MacPherson et al. (2005), Synnott et al. (2010)
	Czf1	- defective filamentation in hypoxia	No	Brown et al. (1999), Chamilos et al. (2009)
	Efg1	- increased resistance to antimycin A (a respiration inhibitor) - hyperfilamentation in hypoxia - defective biofilm formation in normoxia and hypoxia	Yes	Lo et al. (1997), Mulhern et al. (2006), Stichternoth and Ernst (2009)
	Ace2	- defective filamentation in hypoxia - increased resistance to antimycin A	Yes	MacCallum et al. (2006), Mulhern et al. (2006)
	Tye7	- hyperfilamentation in hypoxia - reduced biofilm formation	Yes	Askew et al. (2009), Bonhomme et al. (2011)

*NA, non-available.*

**Table 2:** Phenotypes key regulatory gene null mutants for iron homeostasis.

Organism	Regulator	Phenotype of knock-out mutants	Virulence-related	Reference
*C. neoformans*	Cir1	- hypersusceptibility to phleomycin - defective growth in iron-replete conditions - poor growth at 37°C - loss of capsule formation - altered melanin production	Yes	Jung et al. (2006)
	HapX	- defective heme utilization in culture - transcriptional derepression of iron-dependent pathways during iron starvation	Yes	Jung et al. (2010)
*A. fumigatus*	SreA	- defective growth in iron-replete conditions - hypersusceptibility to phleomycin and oxidative stress - increased siderophore production in iron-replete conditions - hypersusceptibility to amphotericin B and decreased susceptibility to posaconazole and voriconazole in iron-replete conditions	No	Schrettl et al. (2008)
	HapX	- defective growth and sporulation in iron starvation - increased mitochondrial DNA content - decreased resistance to tetracycline - decreased siderophore production - increased zinc sensitivity - transcriptional derepression of iron-dependent pathways during iron starvation	Yes	Schrettl et al. (2010a)
	AcuM	- defective growth in iron starvation - decreased siderophore production	Yes	Liu et al. (2010)
*C. albicans*	Sef1	- defective growth in iron starvation	Yes	Noble et al. (2010), Chen et al. (2011) **
	Sfu1	- defective growth on solid media in iron-replete conditions - transcriptional derepression of iron uptake during iron sufficiency	No	Chen et al. (2011) **
	Hap43	- defective growth in iron starvation - transcriptional derepression of iron-dependent pathways during iron starvation	Yes	Chen et al. (2011), Hsu et al. (2011)
*H. capsulatum*	Sre1*	- increased siderophore production during iron sufficiency - delayed filamentation on *Histoplasma* macrophage medium (HMM)	NA	Hwang et al. (2011)
*B. dermatitidis*	SreB	- reduced growth rate - impaired yeast to hyphae transition by a temperature shift from 37 to 22°C - formation of yellow-pigmented colonies due to defective repression of siderophore biosynthesis in iron-replete conditions	NA	Gauthier et al. (2010)

*NA, non-available.*

* A “knock-down” mutant using RNAi.

Oxygen is an important molecule that has driven a transition of an anaerobic to an aerobic life style in living organisms (Goldfine, 1965; Raymond and Segre, 2006). Uptake of molecular oxygen (O_2_) from the environment is essential for ATP production in cellular respiration (Campbell and Reece, 2002) and a number of biochemical pathways related to biosynthesis of unsaturated fatty acids, tyrosine, sterols, and nicotinic acid (Goldfine, 1965; Raymond and Segre, 2006). It has been reported that both pathogens and host cells are exposed to hypoxia at sites of infection due to decreased tissue perfusion caused by microvascular injury, or increased interstitial pressure with metabolic activities of pathogens and inflammatory cells (Nizet and Johnson, 2009). Although precise oxygen levels at sites of fungal infection have not been studied, certain organs targeted by fungal pathogens such as the gastrointestinal tract (*C. albicans*) and the brain (*C. neoformans*) are known to contain regions of hypoxia (He et al., 1999; Erecinska and Silver, 2001; Sharp and Bernaudin, 2004). In addition, hypoxia was visualized at sites of *A. fumigatus* murine infection in the lung with a chemical hypoxia detection agent, pimonidazole hydrochloride (Grahl et al., 2011). The loss of detectable fluorescence *in vivo* of an *A. fumigatus* luciferase expressing strain despite an increase in fungal burden has been hypothesized to result from hypoxia (Brock et al., 2008). These data suggest that fungal pathogens encounter areas of the mammalian body that are severely limited in oxygen availability.

In human pathogenic fungi, a major transcriptional regulator of the fungal hypoxia response is the sterol regulatory element-binding protein (SREBP). SREBPs are conserved transcriptional activators in many eukaryotes that regulate expression of genes involved in cholesterol biosynthesis in response to changes in cellular sterol levels (Brown and Goldstein, 1999; Osborne and Espenshade, 2009). SREBPs have been observed as key factors in fungal hypoxia and low-iron adaptation, and have been studied in fission yeast *Schizosaccharomyces pombe*, and the human pathogens *C. neoformans* and *A. fumigatus* (Hughes et al., 2005; Chang et al., 2007, 2009; Chun et al., 2007; Willger et al., 2008; Osborne and Espenshade, 2009; Wezensky and Cramer, 2011). Null mutants of the SREBP homologs in *C. neoformans* (Sre1) and *A. fumigatus* (SrbA) display defects in hypoxia adaptation, growth under low-iron conditions, ergosterol synthesis, and susceptibility to triazole antifungal drugs (Chang et al., 2007, 2009; Chun et al., 2007; Willger et al., 2008; Blatzer et al., 2011). SREBP-deficiencies cause significant reductions in fungal virulence, which supports in part the hypothesis that hypoxia adaptation is critical for development of lethal fungal diseases (Hughes et al., 2005; Chang et al., 2007, 2009; Willger et al., 2008; Bien and Espenshade, 2010; Blatzer et al., 2011). In contrast, one of the major fungal pathogens *C. albicans* does not have a clear Sre1/SrbA homolog. Instead, hypoxia adaptation in *C. albicans *is mediated by a zinc finger transcription factor, Upc2 (MacPherson et al., 2005; Synnott et al., 2010). A *upc2* null mutant shows defective growth in anaerobic conditions and hypersensitivity to antifungal drugs ketoconazole and fluconazole, which are similar phenotypes observed in the SREBP deletion mutants in *C. neoformans* and *A. fumigatus* (Silver et al., 2004; MacPherson et al., 2005; Chang et al., 2007; Willger et al., 2008). In addition to Upc2, other transcriptional regulators have been studied with regard to hypoxia adaption in *C. albicans*. A null mutant of *efg1* encoding the APSES family transcription factor displays decreased production of unsaturated fatty acids and increased hyphal formation in hypoxia, which suggests that Efg1 plays a role as an activator of fatty acid desaturation and a repressor of filamentation in hypoxia (Setiadi et al., 2006). Efg1 is also required for biofilm formation in both normoxia and hypoxia (Stichternoth and Ernst, 2009). A putative zinc finger transcription factor, Czf1 interacts with Efg1 by derepressing filamentation that is repressed by Efg1 during growth within matrix, and a null mutant of *efg1* exhibits defective induction of Czf1 (Giusani et al., 2002; Vinces et al., 2006). Disruption of Czf1 causes defective filamentation in embedded conditions (Brown et al., 1999). A transcription factor regulating glycolytic genes, Tye7 is essential for biofilm formation, and ∆*tye7* shows hyperfilamentation in hypoxia a consequence of a reduction in ATP synthesis (Bonhomme et al., 2011). In contrast to Efg1 and Tye7, disruption of a homolog of *Saccharomyces cerevisiae* Swi5 transcription factor, Ace2 required for cell separation results in impaired yeast to hyphae transition in hypoxia (Mulhern et al., 2006). These studies suggest that hypoxia adaptation mediated by Efg1, Czf1, Tye7, and Ace2 is important for virulence-related factors in *C. albicans* including biofilm formation and the morphological transition (Rex et al., 2000; Whiteway and Oberholzer, 2004; Jackson et al., 2007). Indeed, Efg1, Tye7, and Ace2 have been reported to have a role in virulence in *C. albicans* (**Table 1**; Lo et al., 1997; MacCallum et al., 2006; Askew et al., 2009).

Iron is an essential element in all organisms for normal cellular functions including cellular respiration, oxygen and electron transport, as well as synthesis of DNA, amino acids, and lipids (Conrad et al., 1999; Welch et al., 2001). However, iron overload can cause detrimental tissue damage by Haber–Weiss/Fenton chemistry (Galaris and Pantopoulos, 2008). Therefore, a balanced cellular iron concentration is crucial for hosts and pathogens (Hentze et al., 2004). In humans, disturbed iron levels result in anemia and impaired cardiopulmonary function (iron deficiency), and hemochromatosis (iron overload; Andrews, 2000a,b). Importantly, it has been reported that iron overload exacerbates fungal infections such as meningoencephalitis, candidiasis, and zygomycosis probably by enhancing aggressive proliferation of microbes in host tissue (Abe et al., 1985; Barluzzi et al., 2002; Chayakulkeeree et al., 2006). Therefore, iron availability in host environments is important for fungal infection (Weinberg, 1999b).

To acquire iron from an environment, fungi generally utilize four strategies: (i) siderophore (low-molecular mass iron chelator)-mediated uptake of iron, (ii) high-affinity reductive iron assimilation, (iii) uptake and degradation of heme, and (iv) low-affinity iron uptake via metal ion transporters (Kosman, 2003; Jung and Kronstad, 2008). Low-affinity iron uptake and reductive iron assimilation are employed by most fungal species. Most fungal species also produce siderophores but prominent exceptions are *S. cerevisiae*, *C. albicans*, and *C. neoformans*, which nevertheless can take up siderophores produced by other species (Haas et al., 2008). Taken together, there are marked differences in the iron assimilation strategies used by different fungal species.

Fungal iron regulation has been extensively investigated in budding yeast *S. cerevisiae* (Kosman, 2003; Kaplan et al., 2006). Remarkably, the regulators involved in iron regulation are only conserved in closely related Saccharomycotina species (Hissen et al., 2004; Schrettl et al., 2004; Haas et al., 2008) and will therefore not be discussed here unless relevant to mechanisms in pathogenic fungi. Research on iron regulation in pathogenic fungi has been conducted in *C. albicans*, *C. neoformans*, *A. fumigatus*, *Histoplasma capsulatum*, and *Blastomyces dermatitidis* (Haas, 2003; Lan et al., 2004; Jung et al., 2006; Jung and Kronstad, 2008; Schrettl et al., 2008, 2010a; Gauthier et al., 2010; Blatzer et al., 2011; Hwang et al., 2011). Iron-responsive GATA transcription factors are characterized as master regulators in fungal iron regulation and mediate expression of iron-responsive genes (Lan et al., 2004; Jung et al., 2006; Schrettl et al., 2008; Gauthier et al., 2010; Chen et al., 2011; Hwang et al., 2011). Among the pathogenic fungi, details in molecular mechanisms of iron acquisition and homeostasis involving these GATA transcription regulators are particularly well studied in *C. albicans*, *C. neoformans*, *A. fumigatus*, and *B. dermatitidis* (Lan et al., 2004; Jung et al., 2006; Jung and Kronstad, 2008; Schrettl et al., 2008; Gauthier et al., 2010; Chen et al., 2011; Kronstad et al., 2011). In *C. neoformans*, disruption of the GATA factor Cir1 causes increased sensitivity to excess iron, and defects in capsule formation, melanin synthesis, cell wall integrity, and virulence (Jung et al., 2006). Similarly, the *A. fumigatus* GATA factor SreA is required for normal cellular response to iron and oxidative stress, but dispensable for virulence (Schrettl et al., 2008). Disruption of the GATA factor homolog Sfu1 is not essential for a normal growth rate in iron-depleted or iron-replete liquid media, and dimorphic transition in *C. albicans* (Lan et al., 2004). Growth of ∆*sfu1* cultures on solid media appears to be slightly inhibited during iron excess (Chen et al., 2011). *B. dermatitidis* cells lacking the GATA factor SreB fail in the yeast to mold transition and in repression of siderophore biosynthesis in iron-rich conditions (**Table 2**; Gauthier et al., 2010).

In addition to the iron-responsive GATA factor, CCAAT-binding complex (CBC) and a regulatory component interacting with the CBC (the CBC-binding factor) have been characterized as important mediators of iron regulation in *C. albicans*, *C. neoformans*, and *A. fumigatus* (Baek et al., 2008; Homann et al., 2009; Jung et al., 2010; Schrettl et al., 2010a; Chen et al., 2011; Hsu et al., 2011). In *C. albicans* and *C. neoformans*, the CBC-binding factors (Hap43 and HapX, respectively) are required for normal growth in iron-limited conditions. Importantly, deficiency of Hap43 or HapX results in decreased virulence in these species (Homann et al., 2009; Jung et al., 2010; Chen et al., 2011; Hsu et al., 2011). In *A. fumigatus*, ∆*hapX* exhibits impaired growth and amino acid pool composition under iron starvation, decreased production of siderophores such as triacetylfusarinine C (TAFC), and attenuated virulence (Schrettl et al., 2010a). It is noteworthy that regulation of transcription of iron-responsive genes by the GATA factor and the CBC-binding factor are negatively linked in *C. albicans* and *A. fumigatus* (Homann et al., 2009; Jung et al., 2010; Schrettl et al., 2010a). In contrast, HapX positively regulates the expression of the GATA factor, Cir1 in *C. neoformans* (Jung et al., 2010). Intriguingly, HapX may be transcriptionally regulated by the *A. fumigatus* SREBP, SrbA, providing support for a genetic link between hypoxia and adaptation to iron starvation (Blatzer et al., 2011; Linde et al., 2012). A null mutant of SrbA displays a marked growth defect in low-iron conditions and a dramatic reduction in siderophore biosynthesis (Blatzer et al., 2011).

In addition to the GATA factor and the CBC-binding factor, iron regulation in *C. albicans* includes a Cys(6)Zn(2) DNA-binding protein, Sef1, which is exclusively found in this species and acts as transcriptional activator of iron uptake and Hap43 (Chen et al., 2011). In iron-limiting conditions, a null mutant of *sef1* displays a substantial growth defect, which suggests that Sef1 is required for adaptation to iron limitation. *C. albicans* is a commensal pathogen residing in different host environments including the mammalian gastrointestinal tract and bloodstream. Thus, it is perhaps not surprising to find that *C. albicans* possesses distinct mechanisms of iron regulation (Almeida et al., 2009). In contrast to the bloodstream, the gastrointestinal tract provides a relatively iron-sufficient environment. Activation of iron-uptake genes by Sef1 is critical in low-iron conditions like the bloodstream, whereas the GATA factor Sfu1 represses the same set of genes in iron-replete condition like the gut environment. Consequently, Sef1 is required for virulence (representing iron starvation) in a mouse model of bloodstream infection, whereas Sfu1 is dispensable for virulence but promotes gastrointestinal commensalism (representing iron sufficiency) in *C. albicans* (Chen et al., 2011).

Related to the GATA factor and the CBC-binding factor, a zinc cluster transcription factor AcuM has been characterized in *A. fumigatus*. Transcriptional profiling indicated that AcuM activates expression of *hapX* and represses expression of *sreA*. ∆*acuM* shows defective growth in low-iron conditions due to impaired reductive iron assimilation and siderophore production. Importantly, ∆*acuM* shows reduced virulence in mouse models of hematogenously disseminated and invasive pulmonary aspergillosis (Liu et al., 2010). These studies illustrate the observations that fungal iron homeostasis mechanisms are regulated by multiple transcription factors that are often critical for fungal virulence (**Table 2**).

## GENOME-WIDE ANALYSES: HYPOXIA-RESPONSIVE GENES vs. IRON-RESPONSIVE GENES

In addition to transcription factors that regulate responses to hypoxia and iron availability, transcriptome profiling data also suggest a potential coordination of hypoxia adaptation and iron homeostasis. First, hypoxia-responsive genes and iron-responsive genes will be compared to find if hypoxia and iron limitation leads to expression of common gene sets. Second, we will correlate expression of the key regulators for hypoxia adaptation and iron regulation in hypoxia and in different iron levels. Third, chromatin immunoprecipitation (ChIP) data will be included as supportive data for transcriptional profiles that will be discussed. Finally, expression of genes involved in heme biosynthesis in response to hypoxia and iron limitation will be discussed. Due to the biochemical properties, heme is likely a principle connector between iron metabolism and oxygen availability. We hypothesize that regulation of genes responsible for heme biosynthesis might provide clues for interconnectivity in fungal responses to hypoxia and different iron conditions. For parallel comparison of data from different organisms and different stress environments, a range of microarray data cited here is restricted to studies on the major key factors (SREBPs and the GATA factor/CBC-binding factor).

### HYPOXIA-RESPONSIVE GENES REGULATED BY SREBP

Transcriptional profiles from *S. pombe*, *C. neoformans*, and *A. fumigatus* provide a list of genes that are differentially expressed in hypoxia (“hypoxia-responsive genes”) in a SREBP-dependent manner (Todd et al., 2006; Chang et al., 2007; Chun et al., 2007; Blatzer et al., 2011). In *S. pombe*, 68% of genes listed for differential expression (≥2-fold) in anaerobic conditions compared to normoxia (atmospheric levels, 21% O_2_) are regulated by Sre1. These genes are associated with the biosynthesis of ergosterol, heme, and ubiquinone (Todd et al., 2006). In *C. neoformans*, 347 transcripts are differentially expressed in response to hypoxia. The genes encoding these transcripts are involved in metabolism of sterol (“*erg*” genes), heme (*hem13* and *hem3*), sphingolipid/fatty acid, carbohydrate and amino acid metabolism, vesicle trafficking, and ribosomal translation (Chun et al., 2007). *C. neoformans* Sre1 regulates transcription of 100 hypoxia-responsive genes including those involved in ergosterol biosynthesis and iron/copper transport. The iron/copper transport gene set includes genes for uptake of copper (*ctr3*), uptake of siderophore-iron (*sit1*), and reductive iron assimilation such as ferric-chelate reductase (*fre7*), ferroxidase (*cfo1*), and a high-affinity iron permease (*cft1*; Haas, 2003; Kosman, 2003; Chang et al., 2007). The hypoxia-responsive genes involved in iron uptake are also highly induced during iron starvation in *C. neoformans* as expected (Jung et al., 2006). Moreover, the functions of the iron-regulated genes have been characterized. For example, Cfo1 and Cft1 are important for high-affinity iron uptake, utilization of Tf-iron (one type of iron-containing protein in hosts (Hissen et al., 2004)) and virulence (Jung et al., 2009). Deficiency in Sit1 causes abnormal melanin deposition and cell wall structure, but not virulence in a mouse model of cryptococcosis (Tangen et al., 2007). It is unclear to date if these genes are required for hypoxia adaptation in *C. neoformans*.

Two transcriptome analyses have shown that the SREBP homolog in the mold *A. fumigatus*, SrbA, is a principle regulator of hypoxia adaptation (Willger et al., 2008; Blatzer et al., 2011). Microarray data from an early response to hypoxia reveal that 1163 genes (about 12% of genome) are differentially (≥2-fold) expressed in ∆*srbA* compared to wild type. The genes are associated with ergosterol biosynthesis, iron acquisition, glycolysis, oxidative stress resistance, and cell wall biosynthesis. SrbA-mediated regulation of iron acquisition genes including ferric-chelate reductase (*fre7*, a homolog of *C. neoformans fre7*), ferroxidase (*fetC*, a homolog of *C. neoformans cfo1*), high-affinity iron transporter (*ftrA*, a homolog of *C. neoformans cft1*), and siderophore transporter (*sit1*, a homolog of* C. neoformans sit1*) is consistent with Sre1-mediated control of iron-related genes in response to hypoxia in *C. neoformans* (Chang et al., 2007; Blatzer et al., 2011). Notably, as mentioned previously, transcriptional induction of the iron-regulatory CBC-binding factor *hapX*, reductive iron assimilation and siderophore biosynthesis appears to be controlled by SrbA in response to iron starvation in *A. fumigatus* (Blatzer et al., 2011; Linde et al., 2012). Direct regulation of the ergosterol biosynthesis genes *erg11A/cyp51A* and *erg25A* along with the siderophore transporter *sit1* was observed as determined by SrbA binding to the respective gene promoter regions via ChIP-qPCR. Thus, the defect in transcriptional induction of key iron acquisition genes in *A. fumigatus* ∆*srbA* may contribute to this mutant’s growth defects during iron starvation and possibly in virulence (Blatzer et al., 2011).

Overall, in hypoxia fungal SREBPs regulate expression of genes involved in physiological processes associated with oxygen in fungi as well as genes related to iron acquisition and metabolism. Interestingly, more SREBP regulated iron-linked genes in hypoxia are found in pathogenic fungi such as *C. neoformans* and *A. fumigatus* compared to the non-pathogenic fission yeast *S. pombe* (Hughes et al., 2005; Chang et al., 2007; Blatzer et al., 2011). Although we cannot rule out the possibility that different experimental designs and utilized analysis tools affect the number of genes identified in these experiments, this observation may imply that pathogenic fungi have evolved to cope with hypoxia (one of the host environment parameters) more sophisticatedly than non-pathogenic fungi by coordinately regulating iron homeostasis. The different metabolisms of the respective organisms are also important to take into consideration (obligate aerobes vs. facultative anaerobe). Despite the presence of common genes regulated by SREBP in hypoxia, regulation of certain genes differs in a species-specific manner. For example, transcription of a heme biosynthesis gene, *hem13* is highly induced in hypoxia in an SREBP-dependent manner in *S. pombe* and *A. fumigatus* (Hughes et al., 2005; Blatzer et al., 2011). *A. fumigatus* *hem13* has an apparent SrbA DNA-binding motif in its promoter region (data not shown). In contrast, *hem13* expression is highly induced in hypoxia but not affected by Sre1p in *C. neoformans* (Chang et al., 2007). This suggests that fungi utilize both common and distinct mechanisms for hypoxia adaptation besides those governed by SREBPs.

A key regulator of hypoxia adaptation in *C. albicans* that shares many features with the SREBPs is Upc2 (MacPherson et al., 2005; Synnott et al., 2010). Upc2 shares a functional similarity with SREBPs but is not a direct homolog. Similar to SREBPs, Upc2 is highly expressed and proteolytically cleaved in response to sterol-depleted conditions, and eventually transported to the nucleus to activate target genes by binding to a sterol regulatory element (SRE) in the promoter of target genes (Vik and Rine, 2001; MacPherson et al., 2005; Ernst and Tielker, 2009). In hypoxia, 528 genes with different expression levels compared to wild type are regulated by Upc2. Expression of genes responsible for ergosterol synthesis, iron acquisition and belonging to the common in several fungal extracellular membranes (CFEMs) family, which are involved in heme uptake (Weissman and Kornitzer, 2004; Ding et al., 2011) are induced in hypoxia and down-regulated in ∆*upc2* (Synnott et al., 2010). Including iron-linked genes, Upc2 regulates genes similar to those found in *C. neoformans* and *A. fumigatus* transcription profiling data (Chang et al., 2007; Blatzer et al., 2011), which suggests that fungi are able to employ similar mechanisms to deal with hypoxia via different transcriptional regulators.

### IRON-RESPONSIVE GENES REGULATED BY TWO MAJOR IRON REGULATORS

Transcriptional profiling data from studies on the GATA factors (Cir1, SreA, and Sfu1) and the CBC-binding factors (HapX and Hap43) have revealed genes (“iron-responsive genes”) associated with iron uptake, metabolism, storage, and transport in response to iron-replete or iron-depleted conditions in *C. neoformans*, *A. fumigatus*, and *C. albicans* (Lan et al., 2004; Jung et al., 2006, 2010; Schrettl et al., 2008, 2010a; Chen et al., 2011; Hsu et al., 2011). In general, iron starvation induces genes in iron acquisition and represses genes involved in iron-dependent pathways such as respiration, TCA cycle, and heme biosynthesis. In *C. neoformans*, Cir1 regulates transcription of iron-responsive genes positively and negatively. Transcription of genes involved in reductive iron uptake (*ftr1*, *cfo1*, and *cft1*) and laccase synthesis is negatively regulated by Cir1 in low-iron conditions. Expression of siderophore transporters (e.g., *sit1*) are positively regulated by Cir1 in iron-limited conditions. This indicates that Cir1 has a dual role as an activator and a repressor of target genes in response to iron limitation (Jung et al., 2006). In partial contrast, the *A. fumigatus* GATA factor SreA is characterized as a general transcriptional repressor of genes involved in iron uptake during iron-replete conditions. SreA transcript is detectable only in iron-replete conditions. Most of the identified 49 SreA target genes are involved in reductive iron assimilation as well as the biosynthesis, uptake and utilization of siderophores (Schrettl et al., 2008). Similarly, the *C. albicans* GATA factor, Sfu1, represses expression of genes involved siderophore-iron uptake and reductive iron assimilation. Notably, Sfu1 also represses expression of the Cys(6)Zn(2) DNA-binding protein Sef1 that appears to be missing in the other fungal species (Lan et al., 2004). During iron starvation, Sef1 affects expression of 223 genes positively or negatively, and the Sef1-activated genes are related to iron uptake including multicopper ferroxidase (*fet3*), iron permease (*ftr1*), ferric reductase (*cfl1*), siderophore transporter (*sit1*), heme iron uptake receptor (*rbt5*), and interestingly the CBC-binding factor (*hap43*; Chen et al., 2011).

A role of the CBC-binding factors as principle regulators of iron-responsive genes has been studied in *C. neoformans* (CnHapX), *A. fumigatus* (AfHapX), and *C. albicans* (Hap43) (Jung et al., 2010; Schrettl et al., 2010a; Hsu et al., 2011). Under low-iron conditions, CnHapX regulates expression of a broader range of genes than the GATA factor, Cir1, by activating genes encoding siderophore transporters and by repressing genes involved in iron-dependent pathways such as electron transport (Jung et al., 2010). Microarray data in *A. fumigatus* show that during iron starvation AfHapX positively mediates transcription of genes related to metabolism of siderophores, carbohydrates, amino acids, lipids, and protein degradation and uptake. Like CnHapX, AfHapX also acts as a repressor in low-iron conditions, and 34% of the repressed genes are involved in iron-dependent pathways such as respiration, TCA cycle, amino acid metabolism, iron–sulfur cluster biosynthesis, heme metabolism, and cellular detoxification (Schrettl et al., 2010a). In low-iron conditions, the *C. albicans* CBC-binding factor Hap43 also down-regulates transcription of genes associated with iron consumption such as respiration and iron–sulfur cluster assembly.

Overall, to achieve iron homeostasis, fungi employ transcriptional regulators to mediate transcription of iron-responsive genes either positively or negatively. Although there are differences in the target genes in the organisms studied, generally, expression of genes involved in iron uptake (e.g., siderophore transporters, reductive iron uptake factors) is repressed in iron-rich conditions by the GATA factors. In contrast, expression of the repressed genes in iron sufficiency becomes derepressed by the CBC-binding factors in iron limitation. However, additional factors likely remain to be found to fully complete the iron mediated genetic network in pathogenic fungi.

### LINKED GENE EXPRESSION OF THE KEY REGULATORS OF HYPOXIA ADAPTATION AND IRON REGULATION IN FUNGI

The major regulators of hypoxia adaptation (SREBPs and Upc2) and iron homeostasis (the GATA factors and the CBC-binding factors) mediate expression of common target genes (e.g., iron uptake-related genes) in response to hypoxia and iron limitation. An important question is whether expression of these key regulators is related to one another. Interactive gene expression of the GATA factors and the CBC-binding factors has been well studied in *A. fumigatus* (Schrettl et al., 2010a; Blatzer et al., 2011). In this fungus, the expression of the two iron regulators is controlled by a negative regulatory feedback loop (Schrettl et al., 2010a). In low-iron conditions, *hapX* transcription is induced to promote expression of genes responsible for iron acquisition and repress genes involved in iron-dependent pathways, whereas *sreA* transcript levels are not detectable due to repression by HapX. Iron-rich conditions lead to promoted expression of *sreA,* which represses expression of *hapX*, thereby inducing iron-dependent pathways and blocking iron acquisition genes. Intriguingly, in addition to hypoxia, iron starvation also induces expression of *srbA* independent of SreA and HapX in *A. fumigatus* (Blatzer et al., 2011). As a consequence, expression of iron uptake-related target genes of SrbA including *hapX* becomes promoted for adaption to iron limitation. Iron regulation of SrbA is reflected by the fact that a short-term shift from iron limiting to iron-rich conditions decreases the transcript levels of *srbA*. Together, these data suggest that a key hypoxia adaptation regulator and key iron regulators coordinate expression of iron-related genes in response to environmental iron conditions.

**FIGURE 1:**
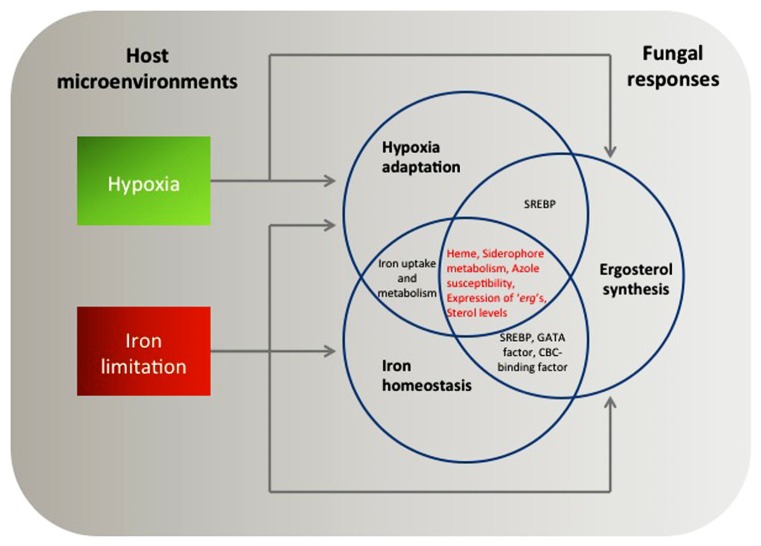
**A putative model of coordinated fungal responses to hypoxia and iron limitation based on what is discussed in this review.** Transcriptional and phenotypic profiles support how fungi adapt to the host microenvironments interdependently. Arrows indicate transcriptional regulation of key mediators of hypoxia adaptation, iron homeostasis, and ergosterol synthesis in response to either hypoxia or iron limitation. Note that heme, siderophore metabolism, susceptibility to azole drugs, expression of “*erg*” genes, and sterol levels are potential common themes controlled during the fungal adaptation processes.

It is noteworthy that both SREBPs and iron homeostasis regulators mediate expression of genes involved in ergosterol synthesis. As mentioned above, all fungal SREBP homologs studied to date (Sre1p and SrbA) and a functional SREBP homolog (Upc2) are associated with transcriptional regulation of ergosterol biosynthesis genes (“*erg*” genes) in hypoxia (Hughes et al., 2005; MacPherson et al., 2005; Chang et al., 2007; Blatzer et al., 2011). Key regulators of iron homeostasis including *C. neoformans* Cir1 and CnHapX as well as *A. fumigatus* AfHapX appear to regulate expression of certain ergosterol biosynthesis genes depending on iron availability (Jung et al., 2006, 2010; Schrettl et al., 2010a). Therefore, we hypothesize that hypoxia adaptation, iron homeostasis, and ergosterol biosynthesis have a tight link to allow fungi to adapt to environmental stresses encountered *in vivo* during human fungal infections (**Figure 1**). Phenotypic analyses to address this hypothesis are discussed in Section “Conclusions and Future Work.”

### A PRINCIPLE CONNECTOR BETWEEN IRON METABOLISM AND OXYGEN AVAILABILITY: HEME

Heme (iron protoporphyrin IX) is found in almost all organisms and critical for electron transfer reactions (Heinemann et al., 2008). It is the major oxygen-binding molecule in eukaryotes and requires iron as a substrate. Heme is an important iron source that some fungi can acquire from the host during infection. Binding of *H. capsulatum* surface to hemin (ferric form of heme, protoporphyrin IX) is induced in iron-limited media (Foster, 2002). *C. neoformans* and *C. albicans* are able to utilize hemin as a sole iron source (Santos et al., 2003). A *C. neoformans* null mutant of the CBC-binding factor, *hapX* shows defective growth in media containing hemin as an iron source, which suggests that the key iron regulator mediates iron utilization from hemin (Jung et al., 2010). However, *A. fumigatus* does not appear to be able to utilize hemin, at least not as an iron source under the tested conditions (Schrettl et al., 2004).

The contribution of heme in adaptation to hypoxia and low-iron environments in fungi is relatively unknown. Heme has been characterized as a major oxygen-sensing molecule in aerobic or anaerobic metabolism in *S. cerevisiae* (Kaplan et al., 2006). Heme biosynthesis requires multiple enzymes including coproporphyrinogen III oxidase (Hem13), protoporphyrinogen oxidase (Hem14), and ferrochelatase (Hem15). In *S. cerevisiae*, in low-oxygen conditions, the rate of heme biosynthesis is decreased, and the absence of heme results in blocking transcription of genes involved in high-affinity iron and copper transport systems (Franken et al., 2011), which suggests a potential role for heme as a connector between oxygen and iron regulation in yeast. During iron starvation, AfHapX negatively regulates expression of heme biosynthesis genes encoding 5-aminolevulinate synthase (HemA), and ferrochelatase precursor (Schrettl et al., 2010a) most likely in order to save iron and to avoid accumulation of the iron-free heme precursor protoporphyrin IX, which is toxic.

Microarray data from *S. pombe*, *C. neoformans*, and *A. fumigatus* show that expression of genes encoding the main heme biosynthesis enzymes is induced in response to hypoxia (Hughes et al., 2005; Chang et al., 2007; Blatzer et al., 2011). In agreement, the cellular contents in heme and iron have been shown to increase during long-term growth in hypoxia in *A. fumigatus* thereby linking hypoxia, iron and heme metabolism (Vodisch et al., 2011). The implication of these findings awaits further mechanistic investigations.

As discussed earlier, hypoxia and iron limitation cause partially similar cellular responses to stress conditions, e.g., up-regulation of iron acquisition. The response is not identical as, e.g., heme biosynthesis and respiration is up regulated by hypoxia in *S. pombe*, *C. neoformans*, and *A. fumigatus* but down-regulated by iron starvation. The explanation for up-regulation of iron acquisition during hypoxia might be related to the increased demand in iron-dependent and heme-dependent proteins, e.g., for respiration and ergosterol biosynthesis to counteract the limited oxygen availability. In contrast, the down-regulation of heme biosynthesis and respiration during iron starvation might support saving iron. These adaptive processes probably reflect pathway prioritizations in order to optimize survival. With data currently available, a full understanding of the interconnections is still missing and most likely not all key players have been identified. One example is that although transcription of *hem13* is induced by SrbA in response to both hypoxia and iron limitation, the regulation appears to be independent of SreA and HapX (Blatzer et al., 2011). Further studies on the biosynthesis and function of heme in pathogenic fungi will be needed to fully address these questions.

## PHENOTYPIC PROFILES OF HYPOXIA ADAPTATION AND IRON HOMEOSTASIS TRANSCRIPTIONAL REGULATORS

### DEFECTIVE GROWTH OF SREBP NULL MUTANTS IN IRON-DEPLETED CONDITIONS

While perhaps the most striking phenotype of fungal SREBP mutants is their inability to grow in hypoxia, it has been reported that loss of SREBP function in fungi affects growth in iron-depleted conditions (Chang et al., 2007; Blatzer et al., 2011). An *sre1* null mutant in *C. neoformans* shows defective growth in iron-limited conditions by iron chelator treatment, and this phenotype is complemented by addition of an iron-source to the media (Chang et al., 2007). Biomass production of *A. fumigatus* ∆*srbA* is compromised particularly during iron-limiting conditions and this defect is rescued in the ∆*srbA* reconstituted strain by expression of *srbA* (Blatzer et al., 2011). Moreover, loss of SreA function in ∆*srbA* can partially restore growth under both low-iron and oxygen conditions (Blatzer et al., 2011). Therefore, SREBPs play a role in coping with both oxygen limitation and iron starvation in fungi.

### A ROLE OF SrbA, SreA, AND HapX IN SIDEROPHORE PRODUCTION IN *A. fumigatus*

Siderophores are ferric iron-specific chelators with low-molecular mass to mobilize iron, and many fungi produce siderophores to maintain iron homeostasis (Haas, 2003; Hissen et al., 2004; Schrettl and Haas, 2011). For example, *A. fumigatus* produces siderophores such as TAFC (extracellular) and ferricrocin (FC, intracellular; Hissen et al., 2004; Schrettl et al., 2004; Haas et al., 2008) and the importance of siderophore production to optimize iron uptake in *A. fumigatus* has been elucidated in several studies (Kragl et al., 2007; Seifert et al., 2008; Wallner et al., 2009; Schrettl et al., 2010b). Molecular genetics of the fungal siderophore biosynthesis and uptake mechanisms are reviewed in detail by Haas (2003).

Transcriptional profiling has shown that expression of certain genes involved in siderophore synthesis and metabolism is regulated by SrbA, SreA, and HapX depending on iron concentrations. Consistently, deficiency in SrbA, SreA, or HapX affects the amount of produced TAFC and FC (Schrettl et al., 2010a; Blatzer et al., 2011). Compared to wild type, loss of SrbA or HapX decreases production of TAFC and FC in iron-limiting conditions, and becomes more severe in a double null mutant of *srbA* and *hapX* (Schrettl et al., 2008, 2010a; Blatzer et al., 2011). In contrast, disruption of SreA increases the production of TAFC and FC under iron-rich conditions compared to wild type. Intriguingly, deficiency of SreA in ∆*srbA* increases production of TAFC and FC compared to ∆*srbA* in both iron-limiting and -sufficient conditions (Blatzer et al., 2011). Given the major role of SrbA in hypoxia adaptation, and transcriptional change of SrbA depending on oxygen levels, it is plausible to conclude that an interdependent mechanism between hypoxia adaptation and siderophore synthesis exists in *A. fumigatus*.

A recent study in *A. fumigatus* supports an enzymatic link between the biosynthesis of ergosterol and siderophores. HMG-CoA reductase (3-hydroxy-3-methyl-glutaryl-CoA) encoded by *hmg1* is a key enzyme involved in ergosterol synthesis and catalyzes the formation of mevalonate. Mevalonate is converted to mevalonyl-CoA by SidI, which is required for TAFC production in *A. fumigatus*. In iron starvation, transcription of *hmg1* is up-regulated and over-expression of *hmg1* causes increased TAFC production (Yasmin et al., 2011). Expression of *hmg1* also appears to be regulated in part by SrbA (Blatzer et al., 2011). Finally, heme is also an essential component of the ergosterol synthesis pathway (Ferreira et al., 2005). Taken together, these studies suggest regulatory links between hypoxia adaptation, iron homeostasis, and ergosterol biosynthesis in fungi.

### EFFECT OF SREBPs AND THE KEY IRON REGULATORS ON PATHOGENESIS

The hypoxia regulators, SREBPs are required for virulence in a mouse model in *C. neoformans* and *A. fumigatus* (Chang et al., 2007; Willger et al., 2008; Blatzer et al., 2011). Disruption of the CBC-binding factors (HapX and Hap43) causes defective virulence in *C. albicans*, *C. neoformans*, and *A. fumigatus* (Jung et al., 2010; Schrettl et al., 2010a; Hsu et al., 2011). The other key regulator of iron homeostasis, the GATA factor, is critical for virulence in *C. neoformans* (Cir1; Jung et al., 2006). The *A. fumigatus* GATA factor SreA and the *C. albicans* GATA factor Sfu1 are dispensable for virulence (**Table 2**; Schrettl et al., 2008). Remarkably, Cir1 carries a single zinc finger compared to the other iron-regulatory GATA factors that contain a double zinc finger. In addition to the structural differences, Cir1 has a dual role as an activator and a repressor whereas most iron-regulatory GATA factors appear to function only as repressors of gene expression (Jung et al., 2006; Schrettl et al., 2008). This is just one of many examples that illustrates the importance of studying the function of potential orthologous genes in their respective organisms.

The implications of coordinated regulation of hypoxia adaptation and iron homeostasis on fungal virulence are poorly understood. As mentioned, inactivation of *sreA* partially cures the growth defects caused by loss of *srbA* including defective growth in hypoxia, iron, and hypersensitivity to triazole antifungal drugs (Willger et al., 2008; Blatzer et al., 2011). However, the ∆*sreA*∆*srbA* strain does not rescue wild type virulence in a mouse model of invasive pulmonary aspergillosis (Blatzer et al., 2011). This is perhaps not surprising given the severe iron limitation found *in vivo* and the modest restoration of hypoxia growth in the ∆*sreA*∆*srbA* strain. Although SREBPs and the CBC-binding factors are crucial for virulence in fungi, it is difficult to conclude whether the defects in adaptation to hypoxia or iron starvation or the combination is responsible for the virulence defect of SREBP-lacking fungi. Future studies will seek to address this important issue.

## A THIRD PLAYER: ERGOSTEROL BIOSYNTHESIS

Ergosterol is the fungal equivalent of cholesterol and inhibition of ergosterol biosynthesis has been a major target for antifungal drug development (Ferreira et al., 2005). Regulation of expression of genes involved in ergosterol synthesis by SREBPs in response to hypoxia has been studied in *S. pombe*, *C. neoformans*, and *A. fumigatus* (Hughes et al., 2005; Chang et al., 2007; Willger et al., 2008; Blatzer et al., 2011). Similarly, the key regulator of hypoxia adaptation in *C. albicans*, Upc2 is also involved in activation of the ergosterol-related genes in hypoxia (Synnott et al., 2010). ChIP analyses show that *A. fumigatus* SrbA and *C. albicans* Upc2 bind to the promoters of ergosterol synthesis genes including *erg25A*, *erg11A/cyp51A* (*A. fumigatus*) and *erg1*, *erg2*, *erg5*, *erg6*, and *erg11* (*C. albicans*; Synnott et al., 2010; Blatzer et al., 2011; Blosser and Cramer, 2012). This indicates that the SREBP regulation of ergosterol-related gene transcription is likely direct. Loss of SREBPs or Upc2 results in a reduction in ergosterol levels, accumulation of sterol intermediates such as C4 methyl sterols, and hypersensitivity to antifungal agents in *S. pombe*, *C. neoformans*, *C. albicans*, and *A. fumigatus*, which suggest that SREBPs are required for normal ergosterol production in fungi (Hughes et al., 2005; MacPherson et al., 2005; Chang et al., 2007; Willger et al., 2008).

Cellular levels and composition of sterols are tightly linked to oxygen availability and in *S. pombe* it seems clear that the SREBP pathway monitors ergosterol levels as an indirect sensor of oxygen (Hughes et al., 2005). When cellular ergosterol levels decrease by treatment of sterol inhibitors, Sre1 proteolytic cleavage increases. Loss of acyl-CoA:sterol acyltransferase, Are2, causes an increase of free ergosterol levels, which results in reduced Sre1 cleavage compared to wild type (Porter et al., 2010). Importantly, ergosterol biosynthesis also involves heme-dependent enzymes (Ferreira et al., 2005) whose transcription alters in hypoxia and in response to different iron levels. Therefore, a critical question is whether ergosterol biosynthesis has an overlapped regulatory mechanism with iron regulation (**Figure 1**).

Major physiological outcomes caused by hypoxia might include depletion of sterol and heme levels in fungal cells. Complex mechanisms of how the sterol and/or heme levels affect regulation of hypoxic induced genes were investigated in *S. cerevisiae* (Davies and Rine, 2006). A heme-activated transcriptional regulator Hap1 regulates expression of *erg* genes by binding at promoters as Upc2 does, and disruption of Hap1 causes reduced ergosterol levels (Tamura et al., 2004). Induction of gene expression by hypoxia in fungi could thus be due to (i) sterol depletion, (ii) heme depletion, (iii) depletion of both sterol and heme, (iv) other unknown factors. In hypoxia, regulation of *erg* expression by Upc2 occurs solely due to sterol depletion. In contrast, hypoxia induction of certain genes including *cox5b* (subunit Vb of cytochrome *c* oxidase) and *anb1* (translation elongation factor elF-5A) takes place due to low heme levels. Expression of *dan1*/*tir1* (cell wall mannoprotein) is induced in hypoxia in response to both sterol and heme levels (Davies and Rine, 2006). Therefore, cellular responses to levels of oxygen, sterol, and heme are at least in part interconnected in *S. cerevisiae*, but remain to be elucidated in human pathogenic fungi.

Transcriptional profiling data in *C. neoformans* and *A. fumigatus* show that the GATA factor Cir1 and the CBC-binding factors CnHapX and AfHapX regulate expression of ergosterol biosynthesis genes in response to iron conditions (Jung et al., 2006, 2010; Schrettl et al., 2010b). A *C. neoformans* null mutant of *cfo1* encoding a ferroxidase required for reductive iron uptake from host Tf shows hypersensitivity to fluconazole and amphotericin B compared to wild type. Interestingly, exogenous heme restores normal sensitivity to the drugs, which suggests that deficiency of ferroxidase decreases intracellular heme levels. Presumably, the reduced intracellular heme levels cause impaired ergosterol biosynthesis in ∆*cfo1* because heme is an essential cofactor in the ergosterol biosynthesis pathway (Jung et al., 2009).

An interesting link between iron limitation and antifungal drug resistance has been observed in *C. albicans*. Treatment of cells using iron chelators results in enhanced susceptibility to the antifungal drug fluconazole (Erg11 target), and the defect is rescued by supplementation of media with iron. Iron deprivation leads to a decreased ergosterol content in the membrane and significantly repressed transcription of *erg11* in *C. albicans* cells (Prasad et al., 2006). As with hypoxia, under low-iron conditions, ergosterol biosynthesis initially decreases in both *A. fumigatus* and *C. albicans* (Prasad et al., 2006; Yasmin et al., 2011). These data suggest that iron conditions in environments influence the biosynthesis of siderophores and ergosterol.

Thus, a close relationship between ergosterol synthesis and cellular iron concentrations exist (**Figure 1**). Despite the limited information available, the data discussed above allow us to speculate that fungi possess linked metabolic processes for hypoxia adaptation, iron homeostasis, and ergosterol biosynthesis. Involvement of heme proteins either directly or indirectly in all three themes could be an example of an interconnected mechanism that remains to be fully explored in human pathogenic fungi. Recent data produced from *A. fumigatus* has given a clue to the presence of the potential interdependency. Inactivation of the GATA factor SreA partially complements hypersensitivity to fluconazole in ∆*srbA* (Blatzer et al., 2011).

## CONCLUSIONS AND FUTURE WORK

Hypoxia and iron limitation are critical host microenvironments that fungi must overcome for successful growth *in vivo* and development of disease. These conditions can alter the abundance of key proteins that are targeted by existing antifungal drugs. Therefore, these two themes have become important emerging research topics in fungal pathogenesis with potential clinical significance. In this review, our main purpose is to understand potential integrated regulatory mechanisms for pathogenic fungi to adapt to hypoxia and iron limitation. Currently available data to address our purpose are mostly produced from genomic and reverse genetic approaches regarding key regulators of hypoxia adaptation and iron regulation in *C. neoformans*, *C. albicans*, and *A. fumigatus*. In the future, more in depth biochemical studies may complement and expand the existing genetic data to better define underlying mechanisms. Results of the transcriptome analyses support the presence of common target genes of the key regulators of hypoxia and iron homeostasis. In addition, phenotypic profiles confirm that the genomic analyses reflect cellular responses to hypoxia and iron limitation in fungi. We also suggest that regulation of ergosterol biosynthesis plays a critical role in these coordinated mechanisms (**Figure 1**).

There are a number of unanswered questions related to the regulatory mechanisms of hypoxia adaptation and iron homeostasis in human pathogenic fungi. For example, how does the coordinated regulation contribute to virulence and responses to antifungal drugs? What unknown players involved in regulation of hypoxia adaption and/or iron homeostasis remain to be identified? For example, as shown in studies on HIF and iron-regulatory proteins in mammals, it is probable that direct regulation among the key regulators/unknown factors exist. One of the studies to address this question could be an investigation of proteins binding to the promoters of the key iron homeostasis regulators using ChIP-seq analysis under various conditions of iron and oxygen stress to fully define the underlying genetic networks. Recent studies elucidating the biofilm and cell wall biosynthesis genetic networks in *C. albicans* present exciting examples of the power of this approach to identify new genes involved in important biological processes (Blankenship et al., 2010; Nobile et al., 2012). It is anticipated that similar and other studies on oxygen and iron availability will provide further evidence for the presence of coordinating regulatory mechanism(s). These data are critical for defining how fungi remodel cellular metabolism against microenvironmental stress encountered during infection. Ultimately, a further understanding of these mechanisms is predicted to yield new therapeutic drugs or strategies to thwart these often-lethal infections.

## Conflict of Interest Statement

The authors declare that the research was conducted in the absence of any commercial or financial relationships that could be construed as a potential conflict of interest.
